# Efficacy of switching from originator adalimumab to biosimilar adalimumab-AACF in patients with rheumatoid arthritis: a 12-month observational study

**DOI:** 10.1186/s42358-025-00480-5

**Published:** 2025-10-10

**Authors:** Fanny Reyes-Neira, Barbara Bayeh, Karina Rossi Bonfiglioli, Nadia Emi Aikawa, Ana Paula Luppino Assad, Renata Miossi, Fernando Henrique Carlos de Souza, Carlos Emilio Insfran, Henrique Ayres Mayrink Giardini, Emily Figueiredo Neves Yuki, Eloisa Bonfa, Carla Gonçalves Schahin Saad, Julio Cesar Bertacini de Moraes, Ana Cristina de Medeiros-Ribeiro, Andrea Yukie Shimabuco

**Affiliations:** 1 Rheumatology Division, Hospital das Clinicas HCFMUSP, Faculdade de Medicina, Universidade de Sao Paulo, Sao Paulo, SP Brazil; 2 Rheumatology Division, Hospital das Clinicas HCFMUSP, Faculdade de Medicina, Universidade de Sao Paulo, Av. Dr. Arnaldo, n° 455, 3o andar, sala 3192, São Paulo, SP Brazil

**Keywords:** Tumor necrosis factor inhibitors, Biosimilar, Originator, Adalimumab, Rheumatoid arthritis, Switching

## Abstract

**Background:**

TNF-inhibitors like adalimumab (ADA) have been vital for managing rheumatoid arthritis (RA) for over two decades. With the emergence of biosimilars, real-life evidence is crucial to confirm their sustained efficacy after switching from originator drugs. This study aims to provide comprehensive evaluation of the clinical outcomes of transitioning from adalimumab reference product (ADA-RP) to the biosimilar adalimumab–AACF (ADA-AACF) in patients with RA over a 12-month period in a real-world analysis.

**Methods:**

This observational study included RA patients who had been on ADA-RP for at least three months before switching to ADA-AACF. Disease activity parameters, including DAS28-CRP, CDAI, SDAI, and CRP-levels, were assessed at baseline (T0) and compared at 6 (T6) and 12 months (T12) following the switch.

**Results:**

Sixteen patients were included, with a mean duration of ADA-RP use of 69.9 months. DAS28-CRP remained stable when comparing T0 to T6 [2.36 (1.92–2.84) vs. 1.83 (1.72–3.16), *p* = 0.988] and T12 [2.36 (1.92–2.84) vs. 2.54 (2.09–2.86), *p* = 0.874], with similar results for SDAI and CDAI (*p* > 0.05). The frequency of low disease activity was consistent during the follow-up: 81.3% at T0; 68.8% at T6 (*p* = 0.617) and 81.3% at T12 (*p* = 0.480) for DAS28-CRP < 3.2. The same pattern was observed for CDAI ≤ 10 and SDAI ≤ 11 (*p* > 0.05). CRP-levels and prednisone doses did not show significant variation (*p* > 0.05) across time points. The biosimilar retention rate was 87.5%, with two patients discontinuing biosimilar before the 12-month mark.

**Conclusion:**

This real-world study demonstrates the feasibility and sustained efficacy of transitioning from ADA-RP to ADA-AACF in RA patients, reinforcing its role in long-term disease management. These findings in an ethnically diverse population contribute to the growing global evidence on biosimilar adoption, supporting their integration into routine clinical practice and expanding treatment accessibility.

## Introduction

Adalimumab is a fully human monoclonal antibody and a TNF inhibitor (TNFi). This biologic class of disease-modifying antirheumatic drugs (DMARD) has revolutionized rheumatoid arthritis (RA) treatment over the past two decades. It is effective in the treatment of adult patients with active RA, who have had an inadequate response to synthetic DMARD (sDMARD), providing reliable disease control and significantly improving long-term outcomes. By selectively binding to tumor necrosis factor-alpha (TNF-α), a central inflammatory cytokine in the pathogenesis of RA, ADA prevents the interaction with inflammatory cell receptors, thereby reducing inflammation and disease activity and preserving patients’ function and quality of life [1, 2].

The advent of biosimilars, biological products highly similar to an already approved reference biological product, has played a pivotal role in expanding access to biological DMARD (bDMARD) following the expiration of originator patents [3–12]. While the efficacy of these medications is well established, their historically high costs have limited patient access, placing a financial burden on healthcare systems. The introduction of biosimilars, including ADA biosimilar, has not only improved cost-effectiveness but also increased treatment availability through competitive market dynamics [12–14].

A growing body of clinical trials evidence supports the safety and comparable efficacy of ADA biosimilars in RA, including data from head-to-head comparisons [3, 5, 6] and trials evaluating switching from originators to biosimilars [3, 6, 10]. These studies have primarily focused in European, North American and Asian RA populations, and have not assessed the performance of the ADA-AACF biosimilar (MSB11022) [15–22].

While some real-world studies have confirmed the impact of the ADA biosimilar in RA [23–29], limitations remain. Some reports did not evaluate the switch from the reference product [23, 24], while others have a short follow-up periods of less than a year [25, 26]. Notably, the only two observational studies assessing the performance of ADA-AACF lacked a specific focus on switching from ADA-RP [23] or analyzed the transitions over just six months [29].

Given these gaps, generating a real-world data on long-term efficacy and drug survival of biosimilar transitions in ethnically diverse populations is essential to further validate their role in clinical practice.

This study aims to conduct a novel evaluation of the clinical efficacy of the ADA-AACF biosimilar (MSB11022) in RA patients transitioning from the reference product, assessing disease activity parameters and drug survival over a 12-month period. By contributing to real-word Latin American data, this study adds to the global understanding of biosimilar adoption and its impact or RA management.

## Materials and methods

### Study design

This is a single-center, longitudinal, observational study conducted in a treatment-inception cohort that evaluated RA patients who transitioned from ADA-RP to the biosimilar ADA-AACF, starting in September 2022, when this biosimilar became available locally. The study assessed data from a prospective database collected through electronic medical records, which included regular clinical and laboratory assessments every three months or at any necessary intervals at a Tertiary Biological Center. To be included in the analysis, patients were required to have been on ADA-RP for at least three months and to have experienced no interruption in treatment at the time of the switch.

### Patient population

Sixteen RA patients aged over 18 years old, meeting the 2010 ACR/EULAR classification criteria [30], and followed at the Rheumatoid Arthritis Outpatient Clinic and the Immunobiological Drugs Infusion Center (CEDMAC – Centro de Dispensação de Medicação de Alto Custo) of the Hospital das Clínicas, Faculty of Medicine, University of São Paulo, were evaluated.

Informed consent to participate in the study was obtained from all participants before initiating biological therapy.

### Data collection

Data regarding RA activity parameters were collected at baseline (T0), 6 months (T6), and 12 months (T12) after the switch from ADA-RP to ADA-AACF. The activity parameters assessed included DAS28-CRP (Disease Activity Score-28 for Rheumatoid Arthritis with C-reactiveprotein), CDAI (Clinical Disease Activity Index), and SDAI (Simple Disease Activity Index), as well as C-reactive protein levels (CRP, mg/L) and prednisone dosage at all three assessment time points. Low disease activity rates for RA was defined according to DAS28-CRP (< 3.2), CDAI (≤ 10), and SDAI (≤ 11) [31]. Information on allergic reactions during the period was also collected.

### Statistical analysis

The results were presented as medians with [interquartile ranges] for continuous variables and compared using a paired T-test or the Wilcoxon test, as appropriate. Categorical variables were expressed as percentage and evaluated using the McNemar’s Test. Statistical significance was considered when *P* < 0.05. Statistical analyses were performed using SigmaStat version 3.1 (2005) and GraphPad/ Prisma Software.

## Results

### Patient characteristics

A total of 16 patients were included in the evaluation of ADA-AACF efficacy six and 12 months after the switch. The group had a median age of 63.1 years (60.2—69.0), with a higher prevalence of female patients (93.5%). Autoantibody positivity was observed in 87.5% (14/16) for rheumatoid factor and 75.0% (6/8) for anti-cyclic citrullinated peptide (anti-CCP). The median duration of prior ADA-RP treatment was 35.4 months (15.7—111.9). The recorded use of concomitant sDMARD was 87.5% (14/16) at the baseline and 85.7% (12/14) at the final assessment. The majority of patients (12/16, 75%) initiated ADA therapy after the failure of at least one prior advanced therapy, with only one patient using it as their first immunobiologic. Conversely, three patients were classified as refractory, having previously undergone at least four other advanced therapies.

### Disease activity parameters

Compared to baseline (T0), DAS28-CRP remained stable at 6 months [2.36 (1.92–2.84) vs. 1.83 (1.72–3.16), *p* = 0.988] and 12 months [2.36 (1.92–2.84) vs. 2.54 (2.09–2.86), *p* = 0.874)]. Similar trends were observed for CDAI and SDAI as demonstrated in Table 1. The assessment of inflammatory markers (CRP) and prednisone dose also remained stable at all-time points (*p* > 0.05) (Table 1).

**Table 1:** Disease activity parameters in RA patients at baseline, 6 and 12 months after switching from ADA-RP to ADA-AACF

	Baseline (*N* = 16)	6 months (*N* = 15)	*P*	12 months (*N* = 14)	*P*
Prednisone dose (mg/day)	0 (0–3.75)	0 (0–1.88)	0.750	0 (0–0)	> 0.999
DAS28-CRP	2.36 (1.92–2.84)	1.83 (1.72–3.16)	0.988	2.54 (2.09–2.86)	0.874
CDAI	5.0 (3.0–8.0)	4.0 (1.0–11.0)	0.855	4.6 (4.0–7.5)	0.928
SDAI	5.3 (4.0–8.2)	4.1 (1.9–12.1)	0.890	6.2 (4.2–8.1)	0.968
CRP (mg/L)	3.0 (2.1–13.9)	3.3 (1.8–9.5)	0.885	3.7 (2.0–5.8)	0.808

Data are presented as median and interquartile range

DAS28-CRP = Disease Activity Score 28 - C-Reactive Protein; CDAI = Clinical Disease Activity Index; SDAI = Simplified Disease Activity Index; CRP = C-reactive protein

### Low disease activity

At baseline, 13 (81.3%) patients had low disease activity for DAS28-CRP, which was maintained at 6 months (11; 68.8%, *p* = 0.62) and 12 months (13; 81.3%, *p* = 0.48). A similar pattern was observed for CDAI ≤ 10 and SDAI ≤ 11 as demonstrated in Table 2. In this analysis, the last observation carried forward was used for the two patients who discontinued the medication during the period.

**Table 2:** Low disease activity frequency in RA patients at baseline, 6 and 12 months after switching from ADA-RP to ADA-AACF

*N* = 16	Baseline	6 months	*P*	12 months	*P*
DAS28-CRP < 3.2	13 (81.3%)	11 (68.8%)	0.617	13 (81.3%)	0.480
CDAI ≤ 10	13 (81.3%)	11 (68.8%)	0.480	12 (75.0%)	> 0.999
SDAI ≤ 11	13 (81.3%)	11 (68.8%)	0.480	12 (75.0%)	> 0.999

Data are presented as number and percentage (%)

DAS28-CRP = Disease Activity Score 28 - C-Reactive Protein; CDAI = Clinical Disease Activity Index; SDAI = Simplified Disease Activity Index; CRP = C-reactive protein

### Retention rate

The drug retention rate at 12 months was 87.5%. Two patients discontinued the use of ADA-AACF before the last evaluation due to refractoriness, while awaiting new therapeutic options. One patient transitioned to a fifth advanced therapy after 9 months on ADA-AACF, while the other switched after 2 months, with an indication for transitioning to the sixth bDMARD.

### Allergic reactions

No allergic reactions were recorded among the 16 patients included in the evaluation.

## Discussion

This study represents a unique experience of switching from the ADA-RP to ADA-AACF biosimilar in RA patients, reinforcing the previously demonstrated disease stability observed with other biosimilars and expanding the evidence that this effect is sustained in real-world long-term scenarios in ethnically diverse populations.

One of the strengths of this study is the 12-month real-world evaluation conducted in a single center, allowing for a systematic analysis of disease activity. The study population was homogeneous, consisting exclusively of RA patients primarily presenting low disease activity, which enabled an accurate assessment of the switch´s efficacy and safety. The main limitation is the modest sample size, which may have resulted in an over or underestimation of certain outcomes. Furthermore, serum drug levels and anti-drug antibodies were not measured, limiting the ability to assess the role of immunogenicity in drug retention.

Evidence on the use of ADA-AACF in inflammatory rheumatic diseases remain quite limited. The only clinical trial involving ADA-AACF in patients with RA confirmed its efficacy compared to the reference drug in European patients over a one-year follow-up period. However, this study did not evaluate the impact of switching between medications [32].

Real-world studies have primarily focused on other ADA biosimilars, such as ABP 501 and SB5, often including patients with conditions beyond RA [25–28] and typically assessing shorter follow-up periods of six months [25, 26]. Moreover, none of these studies included ethnically diverse populations of Latin American. The few reports that evaluated ADA-AACF did not analyze switching [23] or did not exclusively assess patients transitioning from ADA-RP to ADA-AACF, as they included patients who were already using either the reference drug or another ADA biosimilar [29].

Therefore, the present study contributes new evidence demonstrating sustained stability in disease activity parameters over a 12-month follow-up period following the switch from ADA-RP to its biosimilar, ADA-AACF. Using validated and consistent measures such as DAS28-CRP, CDAI, and SDAI, this study provides robust real-world data. The high and persistent rate of low disease activity observed throughout the study aligns with data from the PROPER study [27], which also reported disease stability in European RA patients, with 80.1% and 79.3% of patients maintaining low disease activity at baseline and one year after switching to the SB5 biosimilar, respectively. Moreover, the 87.5% one-year retention rate observed herein study is comparable to historical retention rates for originator biologics and aligns with findings of other studies with similar adherence rates following switches to various biosimilars drugs [5, 7–9, 27, 28] in inflammatory arthropathies. Notably, the PROPER registry [27], reported a one-year retention rate of 72.4% among RA patients who switched from the originator to biosimilar adalimumab-SB5.

The widespread adoption of biosimilars has the potential to significantly reduce treatment costs, enabling broader access to biological therapies without compromising treatment quality [12–14]. Furthermore, clear and informed communication between healthcare professionals and patients can help mitigate potential negative effects associated with the nocebo effect and the transition to biosimilars, ensuring a smoother switching process and greater treatment acceptance [12, 26, 33–35]. Our switching protocol, developed by a specialized medical and multidisciplinary team, included structured patients education on biosimilar equivalence, which may have contributed to the observed outcomes. Expanding real-world evidence on biosimilar efficacy through enhances trust in this strategy among both healthcare professionals and patients.

This real-world study supports the clinical stability and long-term efficacy of switching from ADA-RP to ADA-AACF in RA patients, adding relevant data from an underrepresented Latin American population.

## Data Availability

No datasets were generated or analysed during the current study.

## Abbreviations

ADA: Adalimumab
RA: Rheumatoid arthritis
ADA-RP: Adalimumab reference product
ADA-AACF: Adalimumab-AACF
DAS28-CRP: Disease Activity Score-28 for Rheumatoid Arthritis with C-reactive protein
CDAI: Clinical Disease Activity Index
SDAI: Simple Disease Activity Index
CRP: C-reactive protein
TNFi: TNF inhibitor
DMARD: Disease-modifying antirheumatic drugs
sDMARD: Synthetic disease-modifying antirheumatic drugs
bDMARD: Biological disease-modifying antirheumatic drugs
